# Improved Defect Detection of Guided Wave Testing Using Split-Spectrum Processing

**DOI:** 10.3390/s20174759

**Published:** 2020-08-23

**Authors:** Seyed Kamran Pedram, Tat-Hean Gan, Mahdieh Ghafourian

**Affiliations:** 1 School of Engineering, University of Greenwich, Chatham Maritime, Kent ME4 4TB, UK; 2 Institute of Materials and Manufacturing, Brunel University London, Kingston Lane, Uxbridge UB8 3PH, UK; Tat-Hean.Gan@brunel.ac.uk; 3 Department of Civil Engineering, Brunel University London, Kingston Lane, Uxbridge UB8 3PH, UK; Mahdieh.Ghafourian@brunel.ac.uk

**Keywords:** guided wave testing, signal processing, SSP, SNR

## Abstract

Ultrasonic guided wave (UGW) testing is widely applied in numerous industry areas for the examination of pipelines where structural integrity is of concern. Guided wave testing is capable of inspecting long lengths of pipes from a single tool location using some arrays of transducers positioned around the pipe. Due to dispersive propagation and the multimodal behavior of UGW, the received signal is usually degraded and noisy, that reduce the inspection range and sensitivity to small defects. Therefore, signal interpretation and identifying small defects is a challenging task in such systems, particularly for buried/coated pipes, in that the attenuation rates are considerably higher compared with a bare pipe. In this work, a novel solution is proposed to address this issue by employing an advanced signal processing approach called “split-spectrum processing” (SSP) to minimize the level of background noise and enhance the signal quality. The SSP technique has already shown promising results in a limited trial for a bar pipe and, in this work, the proposed technique has been experimentally compared with the traditional approach for coated pipes. The results illustrate that the proposed technique significantly increases the signal-to-noise ratio and enhances the sensitivity to small defects that are hidden below the background noise.

## 1. Introduction

Ultrasonic guided wave (UGW) testing is one of the advanced Non-Destructive Testing (NDT) techniques and is also known as long range ultrasonic testing. It uses a guided wave signal for testing complex structures such as bars, pipes, etc. UGW testing is broadly used for the inspection of pipelines and has the capability to screen long lengths of these structures in a short time and recognize anomalies (if there are any) from a single test location. A couple of dry transducer arrays are placed linearly around the structure’s circumference in this technology, with some distance from each other, to produce pure axisymmetric (Longitudinal or Torsional) wave modes. It propagates waves within the pipe wall and along the pipe’s main axis. However, the scattering of the waves happens once the waves come across discontinuities in wall thickness. The range of frequencies that are used for UGW testing is normally between 20–100 kHz and the pulse–echo principle is a common approach for UGW testing [1,2,3].

Generally, the attenuation characteristics of UGW testing compared with shear and compression ‘bulk’ wave modes used for conventional ultrasonic testing (UT) are inherently low. The test ranges with guided wave technology, in ideal conditions, are around 50 m in each direction for a bar pipe. However, in real life, most of the pipelines are buried in the ground for safety or aesthetic reasons and, in such cases, non-metallic coatings are commonly applied, which are often viscoelastic in nature, as the primary means of corrosion protection for the pipe. Therefore, the propagation characteristics of the UGW are significantly influenced by the presence of such coatings. These coatings isolate sound energy inside the pipe, particularly when the pipe is buried, which reduces the attenuation ratio of axisymmetric wave modes compared to an uncoated buried/unburied pipe. Numerous factors affect the attenuation such as types of coatings, the material properties of the pipes, test frequency, the coating thickness, etc., or how well the coating is bonded to the pipe. In general, the test range is significantly reduced when using a viscoelastic coating that absorbs the sound energy [4,5,6].

In guided wave technology, short pulses are utilized to minimize the dispersion’s effect and increase the resolution among the features. The excited signal is selected from one of the axisymmetric wave modes, either Torsional or Longitudinal, to produce an axisymmetric wave mode. Moreover, to remove the random noise, the system, as a default, averages the received signal over the repeated test [1,2]. However, due to the shape of the system and the interaction of the signal with features inside/outside of the pipe, mode conversion happens, which generates other wave modes that are mainly dispersive. Xu et al. [7] investigated the mode conversions in the steel plate and stated that mode conversion can occur upon encountering defects within the structure, leading to other wave modes rather than the reflected one. They investigated the group velocity and modal energy of the converted modes using a two-dimensional finite difference time domain approach to measure the scattering field and extract numerical trends. The results show that the apparent group velocities of the converted modes depend on the position of the defect and its severity. They claim that mode conversion can be used to localize a hidden defect. For these types of wave modes, the velocities vary with the frequency, so the energy spreads over time and space throughout the propagation, which makes an analysis of the signal complicated and normal signal processing techniques such as low pass and high pass filtering are not able to remove these dispersive signals that lead to the generation of coherent noise. 

**Figure 1:**
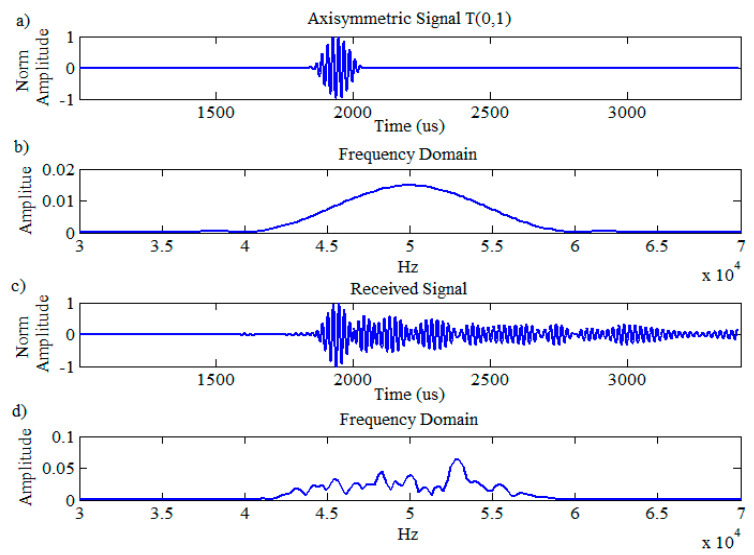
Guided wave excitation signal: (**a**) time; (**b**) frequency domains, received signal; (**c**) time; (**d**) frequency domains.

The typical ideal excitation signal of T(0,1) in the time domain and its response in the frequency domain is illustrated in Figure 1a,b. The received signal which contains the combination of wave modes (T(0,1), its flexural wave mode family F(n,2)) and its frequency response is illustrated in Figure 1c,d, respectively. The flexural wave modes are frequency dependent and dispersive; hence, the received signal, as presented in Figure 1c, is spread out in space and time due to the existence of dispersive modes that leads to generation of background noise. Since the desired reflection signal is based on a single axisymmetric wave mode, the flexural families will be considered as noise that degrades the signal-to-noise ratio (SNR) and spatial resolution of the signal response. Figure 1b,d display the spectra of the ideal reflection and the typical fluctuated received reflection in the frequency domain, respectively. The input pulse is a 50-kHz, 10-cycle, Hann-windowed sine signal. This behavior reduces the sensitivity of guided wave inspection and since it has an equivalent bandwidth to the input signal in the frequency domain, it makes interpretation extremely difficult [5,8].

The main challenge is to identify small features that might be covered by the current noise level, which makes signal interpretation difficult. This is mainly happens due to the complexity of the noise signature that is produced by the existence of undesired wave modes. In particular, the issue of identifying defects in buried and coated pipelines is recognized as a major factor affecting plant availability in the oil and gas market. The dispersion is one of the major sources of coherent noise; thus, it is extremely essential to minimize it somehow to be able to enhance the spatial resolution and identify small defects. One way is to modify the hardware of the system, which is highly costly. Another option would be to investigate the post-processing side of this technology to enhance the signal quality by minimizing the coherent noise and increasing the sensitivity to defects, in particular for buried and coated pipes.

In this paper, an advanced signal processing technique is employed as a post-processing algorithm, known as “split-spectrum processing (SSP)” to minimize the effect of dispersive wave modes at the destination. This technique was already tested for limited scenarios on some random ordinary bar pipes in the lab environment without coatings and achieved promising results [8,9]. This work investigates coated pipelines that, due to their coating, have a considerably higher attenuation compared to the ordinary pipes, and some challenging scenarios are designed for testing the SSP. 

The effect of dispersion on UGW testing has been investigated by researchers over the last few decades, such as Wilcox [10,11] and Sicard et al. [12], to compensate for its behavior, either by reversing the effect of dispersion or employing prior knowledge of its characteristics to map it from the time domain to the frequency domain and then restore it as an undispersed pulse. Dispersion pre-compensation was also considered by Zeng and Lin [13]. They utilized chirp-based narrowband excitation data to utilize the advantage of chirp excitation in order to calculate the propagation distance by employing their previous knowledge of dispersion behavior.

There are some other approaches such as the pulse compression (PuC) method proposed by Toiyama and Hayashi [14], the dispersion compensation (DC) approach proposed by Xu et al. [15,16] and a combination of these approaches developed by Yucel et al. [17,18] to analyze the propagation behaviors of the received signal. Liu et al. [19] investigated an automatic technique for the individual mode extraction of dispersive UGW signals on plates using inversible synchrosqueezed wavelet transform. They tried to obtain a high-resolution time frequency representation and extract its trajectory for each element, employing image processing algorithms. They stated that this approach was successful for automatic mode separation, both for synthesized and experimental UGW signals. The majority of these techniques were successful somehow for highly dispersive signals, but not that successful for non-dispersive wave modes such as axisymmetric ones or the wave modes that have little dispersion such as lower-order flexural wave modes.

Moreover, several post-processing algorithms such as wavelet de-noising and cross-correlation were also considered by Mallet et al. [20] for reducing the effect of dispersion, but the results show that these methods were not successful on UGW testing. This was mainly due to the approach of these techniques, in that, in both cases, any signals smaller than the defined threshold were removed regardless of whether they were actual signals or not. 

Furthermore, the SSP algorithm was investigated for use in non-destructive testing, mainly for conventional UT, to enhance the signal quality [21,22,23]. The results illustrated some enhancements in terms of the grain scattering that occurs when the received signal splits into a set of sub-band signals with different center frequencies. It has been claimed that this enhancement is achieved only when the appropriate filter bank parameters have been identified for the SSP method in UT inspection. According to their theoretical approach, there is some discrepancy in terms of the value of a parameter that changed the result slightly. This was mainly because of using a Gaussian function for filtering rather than a Sinc function due to its simplicity, while the design was based on Sinc function.

The SSP method, in conjunction with an order statistic, was studied by Saniie et al. [24] to enhance the flaw-to-clutter ratio of noise. Although the results show some enhancements where there was enough separation between them, their performance is worsened with undesirable information. 

Additionally, a modified version of SSP called the complex-plane SSP technique was proposed by Rubbers and Pritchard [25], which enhanced the SNR in traditional UT testing by employing additional mathematical information. Then, they reviewed the SSP algorithm [26] and claimed that the use of this approach is limited in practice due to the lack of linearity of the signal’s amplitude; hence, it cannot be used for measuring flaws. SSP, in combination with an order statistic approach, was developed by Syam and Sadanandan [27] to minimize the effect of reverberation by utilizing a wideband signal in conventional UT. Although it has been claimed that they were successful in identifying flaws, their technique is only verified for a synthesized signal and further validation is required.

A new filter bank strategy was proposed by Rodriguez et al. [28,29] by introducing variable bandwidth filters, where the filters are equally spread out in the frequency and their energy gain equalized. The results show that the frequency multiplication (FM) method obtained the highest resolution by reducing the required number of filter bands and the system complexity when used with the proposed filter bank design compared to other approaches. However, it has been claimed that further investigation is required for this approach as it is not evaluated for highly dispersive models, non-stationary structures, or scenarios with a different type of defect.

It has been shown throughout the literature that SSP application could be implemented successfully if appropriate filter bank parameters are selected. These successes were mainly achieved for conventional UT inspection in terms of SNR enhancement. However, these values were soon found not to be suitable for UGW inspection. This is mainly due to the existence of a combination of wave modes that operate in the kHz range (e.g., axisymmetric and non-axisymmetric) with different velocities. In order to find an appropriate parameter of SSP for use in UGW inspection, some parametric studies were carried out in previous works [9,30]. The results presented revealed that the optimum parameters were identified for synthesized signals and ordinary bare pipes in a limited trial in the lab that significantly enhanced the signal quality in terms of spatial resolution and SNR.

In this work, the intention is to identify the optimum parameters of SSP to enhance the signal quality and increase the inspection range for more challenging scenarios where the pipe is partially coated and the attenuation compared to the ordinary pipes is quite high. Therefore, two laboratory experiments were carried out on two eight-inch pipes (schedule 40) to evaluate the proposed method. Both pipes were partially coated with Denso Tape, Winn & Coales International Ltd., which is one of the most common coating materials (spiral wrap) utilized in the industry for coating due to its simplicity, as it can be easily applied over a pipe. 

A Teletest Focus+ unit was employed for the experiment, which is one of the commercially available tools in the market for the inspection of tubular structures [31]. The unit was used for data collection to excite and receive the signal. The pulse–echo technique was also used, where sound was transmitted along the axial length of the pipe to inspect inaccessible areas that are not able to be inspected by conventional methods. The results show that the proposed SSP approach enhances the quality of the signal in terms of SNR by roughly 40 dB compared to conventional means, which is a great success in terms of UGW testing to increase the inspection range and enhance the sensitivity of defect detection.

This paper is structured as follows: the application of split-spectrum processing (SSP) and its implementation are described in Section 2. Section 3 gives details about the selection of SSP parameters. Section 4 and Section 5 provide the lab experiments of the proposed method, followed by field data analysis. Section 6 provides the discussion, and Section 7 concludes the paper.

## 2. Split-Spectrum Processing

**Figure 2:**
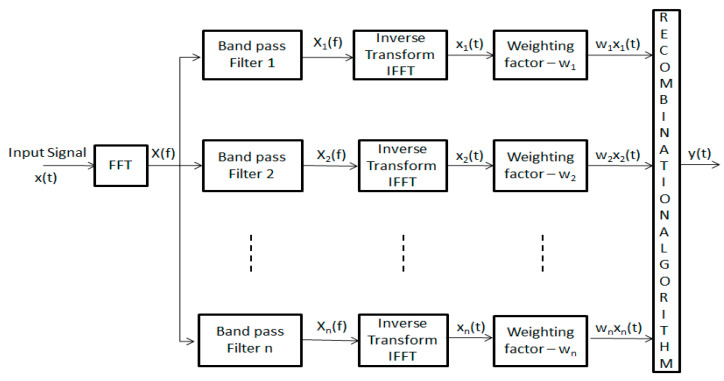
Split-signal processing (SSP) block diagram.

This method splits the spectrum of the signal to create a set of sub-band signals at different center frequencies by employing a bank of band-pass filters. These sub-band signals are subjected to several non-linear processing procedures to produce an output signal. The operation of the SSP method is presented in Figure 2, where the input signal, x(t), is transformed into the frequency domain, X(f), to be filtered, by employing a set of band-pass filters, and inversed back into the original domain. Then, each element is normalized by a weighting factor where each set of signals is divided by its maximum values in the time domain before the recombination algorithms are applied. Finally, all the individual non-linear signals are added together, employing one of the recombination algorithms to yield the output signal, y(t).

As previously mentioned, the velocity of dispersive wave modes varies with frequency, so their components also vary through the sub-bands, whereas, for the non-dispersive wave modes, the components stay unchanged. This behavior is used in SSP method to suppress sections of the signal that fluctuate along with the bandwidth, hence minimizing the effect of dispersive wave modes. 

### 2.1. SSP Recombination Algorithms

There are many approaches to combine the filter bank values to produce an output signal [8,30]. The Polarity Thresholding (PT) algorithm is selected for this exercise as it has shown in previous works that it gives the highest enhancement in terms of spatial resolution and SNR for ordinary pipes without distorting the signal [9]. The PT algorithm can be calculated as:

(1) $$\begin{array}{lll} y\left[m\right]\;=\;x\left[m\right]\; & if\;all\;x_{i}\left[m\right]\;>\;0 & i\;=1,\;\ldots ,\;n\\ y\left[m\right]\;=\;x\left[m\right] & if\;all\;x_{i}\left[m\right]\;<\;0 & i\;=1,\;\ldots ,\;n\\ y\left[m\right]\;=\;0 & for\;all\;other\;values\; & \end{array}$$

where y is the result achieved after the processing of the signal at m, x_i_[m] is the sub-band signal, x[m] is the input signal, and n is the number of filter bands. The signal is analyzed at each sample time for the sub-band signals and if the sign of the samples is the same (either negative or positive), the input signal stays unaffected and passes to the output, whereas if the sign is changed at any of the sample times, then the output becomes zero. Hence, this approach only passes the time samples when the polarity (sign) is unchanged, which means it is not affected by the frequency and only removes the highly frequency-dependent signals. Notably, the noise amplitude needs to be lower than the actual signal’s amplitude, otherwise it changes the signal’s sign. 

### 2.2. Implementation of SSP

The literature states that the filter bank parameters of SSP have a crucial role to enhance the signal quality in terms of spatial resolution and SNR. These parameters, illustrated in Figure 2, are fully explained in previous works [2] and contain (i) filter separation (F), (ii) sub-band filter bandwidth (B_filt_), (iii) the number of filters (N), (iv) filter crossover point (x), and (v) total bandwidth for processing (B). As mentioned earlier, the optimum parameters have been identified for the ordinary pipeline in a limited trial and, in this work, the aim is to validate the proposed method for more complex scenarios where the pipes are partially coated by Denso Tape (Winn & Coales International Ltd.).

The Teletest unit has its own software and A-scan for inspection; however, to implement the proposed method, a set of code was written and developed in the MATLAB environment, which received the unprocessed row data as input signals and transferred them into the frequency domain for post-processing. Gaussian band-pass filters were employed to filter the signal by multiplying their Fourier transform by the Gaussian window to produce a set of sub-band signals, each of which had its own lower $f_{l}$ and higher $f_{h}$ cut-off frequencies, which were calculated as follows:

(2) $$f_{l_{n}}=\{\begin{array}{ll} f_{min}-\frac{B_{filt}}{4} & n=1\\ f_{l_{n-1}}+F & n=2,3,\;\ldots N \end{array}$$

(3) $$\;\;\;\;\;\;\;f_{h_{n}}=\;f_{l_{n}}+\;B_{filt}\;\;\;\;\;\;\;\;\;\;\;\;\;\;\;\;\;\;\;\;\;\;\;\;\;\;\;n=1,2,\;\ldots N\;\;$$

where N is the number of filters, $f_{min}$ is the lower cut off frequency of the whole bandwidth, F is the filter separation, and $B_{filt}$ is the sub-band filters. Moreover, f_l1_ is the lower cut off frequency of the first sub-band that requires us to start from the beginning of the signal. The sub-band signals are then added together using the PT recombination method to produce the signal’s output.

## 3. Selection of SSP parameters

The parameters that were employed for the traditional UT were not suitable for guided wave testing. This was due to the narrow bandwidth and long duration of the guided wave signal, which runs in the kHz range, whereas the conventional ones run in the MHz range. The most common filter separation calculation in UT led to a huge quantity of sub-band filters [21,22,23] which require either a narrow bandwidth or a large overlap. The signal responses might be lost due to the selection of narrowband filters, whereas large overlaps between the sub-bands led to the outcome of each one becoming highly correlated, which could compromise the performance of the proposed method at minimizing the background signal.

**Figure 3:**
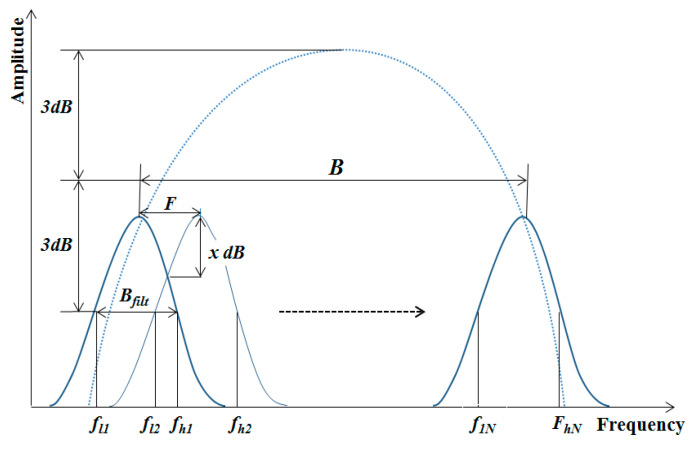
SSP filter design.

To identify the optimum parameters of the proposed SSP method for UGW testing, a brute force search algorithm was employed across the SSP parameters, i.e., the number of filters (N), the filter crossover point (x), the filter separation (F), the sub-band filter bandwidth ($B_{filt}$), and the total bandwidth (B). The full details of these parameters were already discussed in previous papers [5,9]. In brief, these parameters are independent of each other, as illustrated in Figure 3, so if one value changes, the other values are also required to change. Hence, the optimum values need to be identified in parallel. These values were first inspired by the values employed for traditional UT, which provides a suitable range that can be used for different pulse length signals or different frequencies. It is claimed that the processing bandwidths will be increased when choosing higher frequency signal or shorter pulse lengths.

For instance, the number of filters (N), according to Figure 3, can be calculated by dividing the total bandwidth (B) by the value of the filter separation (F) as shown below:

(4) N=B/F +1

In addition, the total bandwidth needs to be selected somehow so that the signal’s reflection from real features within the structure stays constant while the reflection from dispersive wave modes varies. Note that if the bandwidth selected is unsuitable, then either some features of reflection will be lost, or some dispersive wave modes will not be removed, which, either way, reduces the spatial resolution. To reduce the effect of dispersive modes, narrowband waveforms up to 10 cycles are utilized as the excitation signals in UGW testing using Hann-windowed sine waves.

Many researchers suggested [3,21,22,23] that the value of sub-band filters should be set around four times the values of filter separations. It should be noted that selecting the wrong value could lead to a reduction in the temporal resolution as the feature’s reflections will spread out in space/time and cover one another. It was found that [5,8] the 3dB cut-off frequency from the center frequency of the input signal could be employed as the band-pass filters in SSP filter design. Note that the overlap between filters needs to be selected accordingly as it affects the correlation between them, which could either cause the signal of interest to be lost or add noise. Hence, overlap needs to minimize the correlation among the background noise in adjacent sub-bands without losing information.

In terms of filter separation, it was claimed by Karpur et al. [23] that appropriate spectral separation could be obtained by utilizing the frequency sampling theorem. Hence, the SSP filter separation could be calculated as:

(5) F=1/T

where F is the sampling frequency and T is the duration of the input signal. Note that this is based on Sinc function; therefore, the actual value needs to be modified slightly as the Gaussian filter is utilized in this work for its simplicity instead.

As shown in Figure 3, a part of the frequency spectrum of the unprocessed signal was chosen as the total bandwidth (B) that was fed into the system and the values of the rest are varied to identify appropriate values for them. Moreover, the value of the sub-band filter was selected to be a portion of the total bandwidth able to apply for any UGW signal. The SNR of the signal response was measured as a quantified performance to identify the optimum values of SSP parameters. The main concern was to preserve all the axisymmetric features and minimize the coherent noise reflection as much as possible. As a result, the optimum parameters were identified with the following values: (i) 99% of the total bandwidth, (ii) sub-band filter values equal to the total bandwidth divided by 11, (iii) a filter crossover of 1 dB, and (iv) a filter separation equal to the sub-band filter values divided by 1.5.

Consequently, the proposed values were defined as the optimum parameters of SSP for a 10-cycle Hann-windowed UGW signal with center frequencies of 30 kHz and 35 kHz for an eight-inch pipe. These parameters were subsequently utilized in this work for processing the experimental and field data analysis.

In addition, to estimate the attenuation rate, the relevant acoustic properties of the Denso Tape (Winn & Coales International Ltd.) as a coating material is studied to estimate the realistic values of the attenuation rate, in a similar manner to the study carried out in [6,32]. The attenuation of the axisymmetric T(0,1) wave modes, which were employed as the excitation modes, was measured. A trial and error method was employed to extract the material properties by adjusting the properties of the material to obtain a suitable result between the theoretical approach and the measurements for different frequencies. Hence, 3dB/m has been found as an appropriate attenuation rate for the Denso Tape coating and its steps are described in [32]. It is found that its performance heavily depends on the thickness of the coating as well as the coating acoustic properties. This information could be utilized to identify and optimize testing conditions such as selecting the frequency range where the attenuation is less effective.

Moreover, the attenuation rates give rise to a wide range of amplitudes in the signal response across the examination length, which is potentially several meters long. As an example, if the attenuation rate of an ordinary pipe is 0.2 dB/m, an ultrasonic signal with an initial value of unity after 10m propagation will have an amplitude of 0.8. The 3 dB/m attenuation rate is obtained for the coated pipe of the T(0,1) mode; hence, the amplitude is 0.03, whereas, for 6 dB/m, the amplitude will be 0.001, i.e., 1/1000 of the initial amplitude.

## 4. Lab Experiments

To validate the SSP approach for minimizing the effect of undesired wave modes in UGW testing and improve the signal quality, two experiments were carried out in the lab on two similar eight-inch pipes. These pipes were partially coated with Denso Tape (Winn & Coales International Ltd-UK) to simulate the transition from an unburied to a buried part in order to demonstrate its effect. A Teletest device [31] was employed for these experiments using the pulse–echo technique to excite and receive the UGW signal and gather experimental data. The signal was excited/received utilizing a ‘3 Ring Torsional’ Teletest unit to transmit a 10-cycle, Hann-windowed modulated tone burst of T(0,1). The sampling frequency was set to 1MHz and the ring spacing was 30 mm between transducers.

After gathering the data, the proposed SSP technique was applied to them as a post-processing approach to enhance the spatial resolution and increase the SNR of the signal’s response by minimizing the effect of dispersive wave modes. To make the comparison easier between the SSP result and the conventional one, some codes were written in the MATLAB environment to read, analyze and present their results in the same format. Furthermore, the normalized values of amplitudes are presented in the figures to make the comparison easier. Moreover, Hilbert transform was employed in this work to demonstrate the absolute value of a sinusoidal signal both for the SSP and conventional approaches. Therefore, the envelope of the waveforms is displayed to make the comparison easier.

### 4.1. Experiment #1: Pipe A

**Figure 4:**
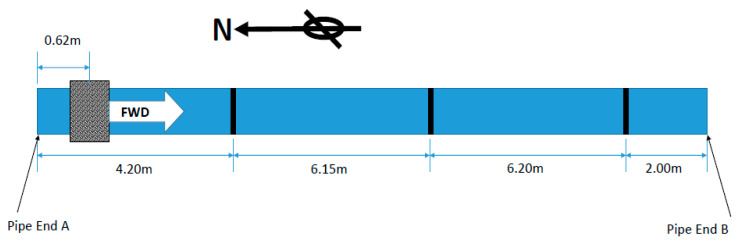
Experimental setup of Pipe A. The tool position is 0.62 m from the end of Pipe A and the welds are illustrated as black lines.

The pipe under investigation (Pipe A) in this exercise was a nominal eight-inch, eighteen-meter long steel pipe, with a wall thickness of 8.28 mm and an outside diameter of 219.08 mm. Figure 4 displays the experimental setup where the Teletest collar was placed at 0.62 m away from the near pipe end on the left-hand side. This was the baseline setup of Pipe A before coating.

**Figure 5:**
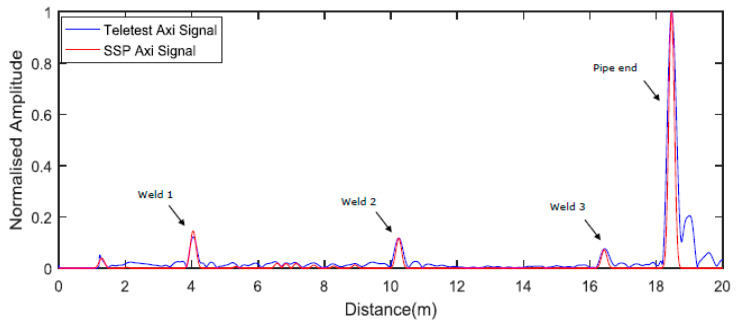
Baseline axisymmetric and SSP signal without coating for 27 kHz.

Figure 5 illustrates the result for the Teletest axisymmetric wave mode (blue trace) and the proposed SSP technique (red trace) for the 27-kHz baseline before coating. As can be seen, the weld reflection amplitude is much less than the pipe end reflection amplitude, as expected in any uncoated pipeline inspection (20%). Note that the SSP parameters were selected randomly for this exercise. Therefore, some background noise (coherent noise) still appears in the output signal. However, the SSP result is much less noisy compared to the unprocessed Teletest response.

#### 4.1.1. Coating the Pipe

**Figure 6:**
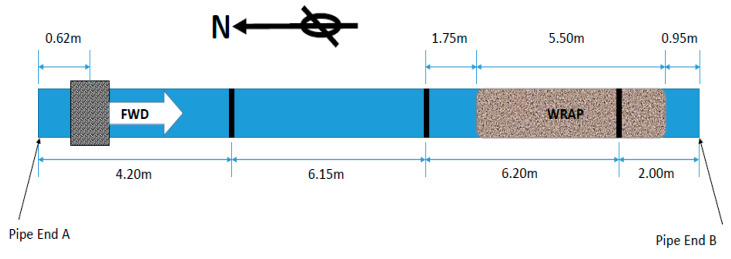
Experimental setup of Pipe A after coating.

As mentioned earlier, the Denso Tape is employed for this experiment due to its simplicity of application on a pipe as a spiral wrap for experimentation to partially coating the pipe under investigation, as illustrated in Figure 6.

**Figure 7:**
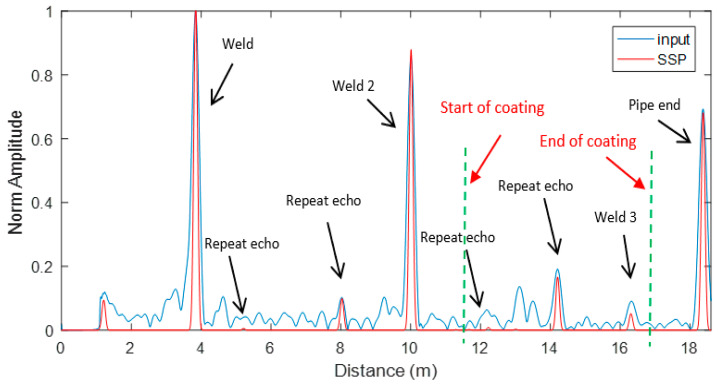
Unprocessed Teletest axisymmetric signal (blue trace) and SSP (red trace) signal with the optimum SSP filter bank parameters for 30 kHz.

The main aim of using the SSP application was to increase the signal quality by preserving the axisymmetric features and minimizing the effect of background noise as much as possible. Figure 7 shows the result of Teletest (blue trace) and the results of the proposed SSP method (red trace) with the optimum parameters for the T(0,1) wave mode, 10-cycle Hann-windowed excitation signal with a center frequency of 30 kHz. The result shows that the proposed system removed all the background noise (coherent noise) in the whole length of the signal and preserved all the reflected echoes from the axisymmetric signals. Note that the optimum SSP parameter values were identified by employing the brute force search algorithm, as described earlier, by measuring the SNR of the signal response using the following calculation:

SNR = 20 log_10_(S/N) (6)

where N is the RMS value of the coherent noise area around the third weld and S is the reflection of the peak value for the third weld. As illustrated in Figure 7, both peaks appeared in the coating area. The results prove that the proposed technique with the optimum parameters is successful in enhancing the signal quality and increasing the SNR by approximately 40 dB compared to the conventional approach without distorting the reflection signal. Note that the signals at 8 m and 14 m are the reflected echoes from the first and second welds, respectively, due to the multiple reflections.

#### 4.1.2. Creating a Defect 

**Figure 8:**
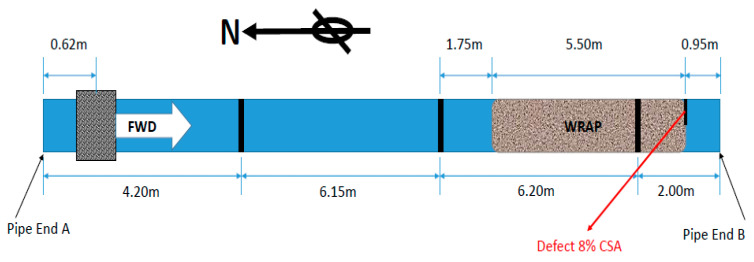
Creating an 8% cross-sectional area (CSA) saw cut defect on Pipe A.

In the next stage, to validate the proposed approach, a saw cut defect with an 8% cross-sectional area (CSA) was cut mid-way between the third weld and the end of Pipe B, right after the coating area, as displayed in Figure 8. 

**Figure 9:**
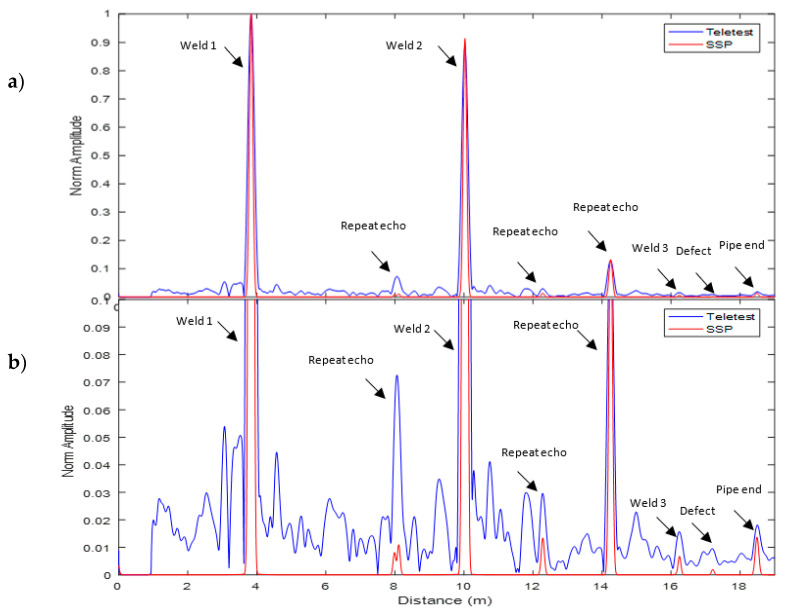
Unprocessed signal (blue trace) vs. SSP (red trace) (**a**) the whole length of the trace (**b**) zoom-in the whole length of the trace.

The Teletest unit was used to excite and receive the signal. The received signal was then post-processed, employing the proposed SSP technique, and compared with the conventional technology. This result is presented in Figure 9, where the unprocessed Teletest data (blue trace) and SSP results (red trace) for the whole length of the trace are illustrated in Figure 9a. Figure 9b displays the zoomed-in results of the same trace. The results demonstrate that the conventional technique is not able to remove the effect of dispersive wave modes; hence, it contains a continuum of coherent noise which cause the defect to be hidden below the noise level. On the other hand, the SSP approach successfully removed the coherent noise throughout the trace length entirely, while it preserved all the axisymmetric features from the real reflectors. This signal includes the echoes from the first and second welds, which have high amplitudes. As a result, an inspector can easily identify a defect using the proposed SSP technique, whereas it was impossible to recognize it with the conventional method.

Moreover, the SNR is calculated using Equation (6) to quantify the enhancement achieved by the proposed method, where N is the RMS value of the background noise around the defect and S is the peak value of the defect’s reflection. It was found that the SNR around the defect increased approximately by 40 dB without distortion in the signal.

### 4.2. Experiment #2: Pipe B

**Figure 10:**
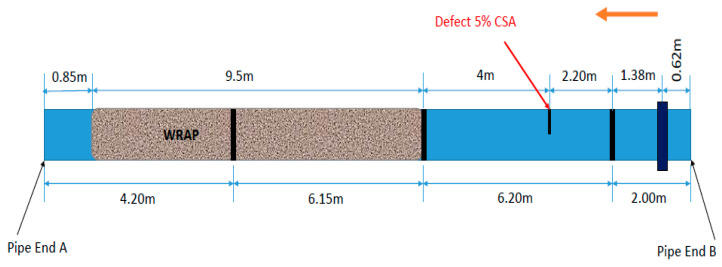
Set-up for Pipe B, including wrapping and a 5% CSA defect. The welds are illustrated as black lines.

In this experiment, the pipe geometry and size were the same as the previous experiment. As shown in Figure 10, the only difference is the length of coating (wrapping) which was started at 0.85 m from the pipe end A for the length of 9.5 m. The Teletest unit is placed at 0.62 m from the pipe end B and a 5% CSA saw cut defect is produced between the first and the second welds as demonstrated in Figure 10.

**Figure 11:**
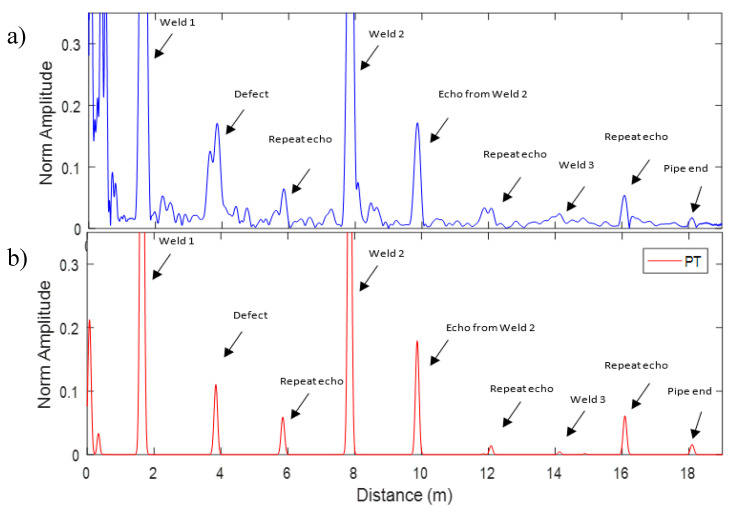
Pipe B result for (**a**) unprocessed Teletest data, (**b**) SSP result for the whole length of the trace.

The result of Teletest approach and SSP method with the optimum parameters is illustrated in Figure 11 for the whole length of the pipe B. Figure 11a indicates that although the Teletest system can identify the defect, the received signal is quite noisy and the third weld is almost hidden on the noise level. On the other hand, the SSP result in Figure 11b illustrates that the proposed method not only cleared the entire signal from any background noise, also preserved all the real features reflections for the whole length of the pipe under inspection. 

**Figure 12:**
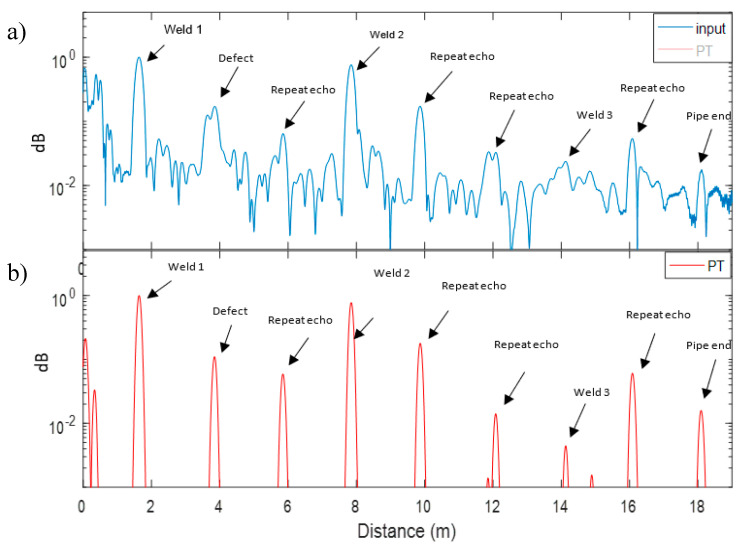
Pipe B log results: (**a**) unprocessed Teletest data, (**b**) SSP result for the whole length of the trace.

Furthermore, Figure 12 shows the unprocessed Teletest (a) and SSP (b) results in log scale respectively. It is clear that the SSP result in Figure 12b is much more useful to identify any features in the pipe as it has removed the coherent noise signal entirely.

## 5. Field Data Analysis

In this section, two sets of field inspection data are considered to validate the proposed SSP technique. Teletest technology is utilized to collect data with the narrowband excitation approach. Then the received signal is inspected by a trained UGW inspection personnel to recognize defects such as erosion or corrosion during the inspection using the conventional approach. This result is then compared with the proposed post-processing SSP method with optimum parameters using the PT recombination algorithm. The PT algorithm is claimed to be the best algorithm in terms of SNR improvement in guided wave testing [9,30]. A key point of this section is to demonstrate how the SSP method can boost the spatial resolution and SNR of the received field data compared to the traditional approach achieved with Teletest technology.

**Figure 13:**
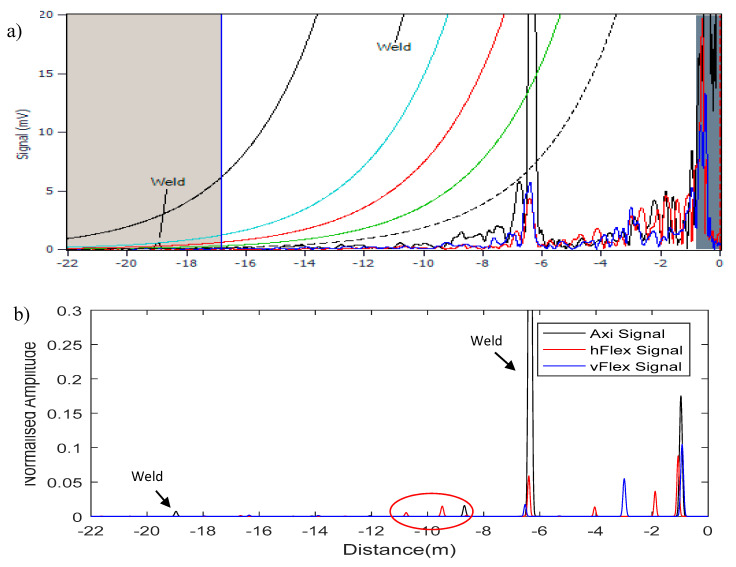
Field data #1; (**a**) Teletest data; (**b**) SSP result.

**Figure 14:**
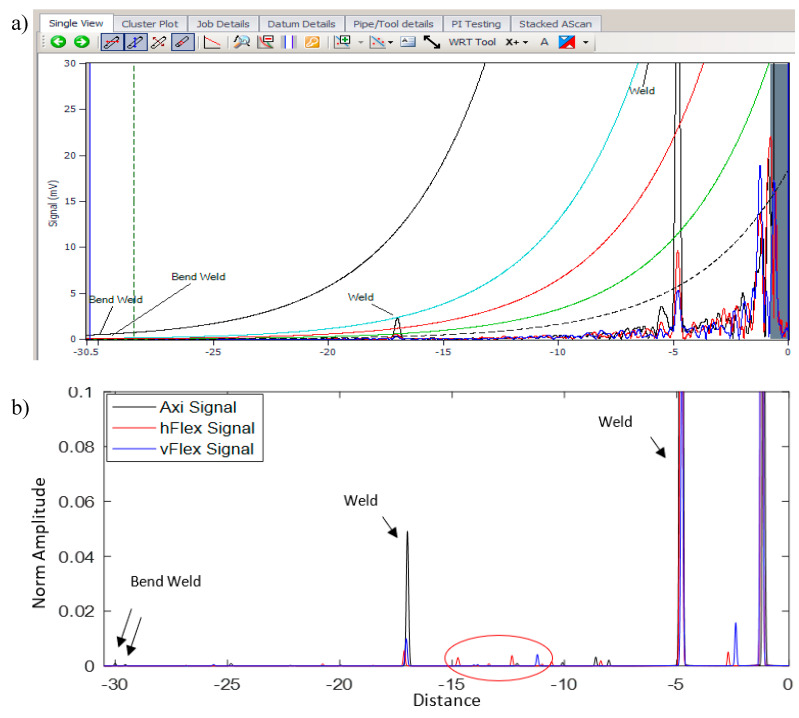
Field data #2 (**a**) Teletest data, (**b**) SSP result.

Figure 13 and Figure 14 illustrate the linear A-scan in the distance domain (*x*-axis) and the power amplitude (*y*-axis) of the reflection’s signal that the operator utilized to analyze the data and decide whether there were any features within the structure. The black trace displays the axisymmetric T(0,1) wave mode; the blue and red traces present the vertical and horizontal flexural responses of F(1,2) in terms of cross-section displacement, respectively. In addition, distance amplitude correction (DAC) was employed in the Teletest device to set the reference signals for measuring the sensitivity of the scan and categorize the defects that the details are explained in [1].

Figure 13a shows the conventional result of the field data (#1). The majority of the pipe was placed under the ground. It can be seen from the result that apart from the welds that are already identified, it is highly unlikely that the inspector could identify any other features on this pipe. However, after digging up part of this pipe, it has been reported by the client that there were some small features on the pipe around −8 m to −10 m from the Teletest collar location which was unrecognized by the conventional method. The SSP technique applied to this data and the result presented in Figure 13b. The results confirm that the SSP technique flagged a few features in the same area reported by the client.

The same scenario occurred for the second set of field data (#2). The results of the conventional technique and SSP method are demonstrated in Figure 14a,b, respectively. In these data, two welds and two bend welds were identified by both methods. In addition, the SSP results claimed that there were some small features around −10 m to −15 m from the tool location that needed to be investigated further. In the report generated by the client after digging up the pipe, it was mentioned that some features were identified on that region, which could confirm the SSP result flagging up some small peaks in that region. 

However, this is a limited trial of field data analysis to confirm that the SSP method has the capability to improve the spatial resolution, SNR and increase the inspection range for pipeline inspection. In order to fully validate this method, more field data analysis is required.

## 6. Discussion

The results obtained within this work prove that the SSP method, with optimum parameter values, has great potential to enhance the UGW signal’s quality by minimizing the effect of background signals (coherent noise) in the signal’s response, primarily caused by the existence of undesired wave modes. Defect identification is one of the primary benefits of using UGW testing for the inspection of pipelines, but this method is considerably affected by the presence of coherent noise. 

Hence, a technique is required in order to address the issue of such noise in UGW testing. As a result, the SSP technique is proposed to address this by minimizing the effect of dispersive wave modes. According to the literature, the outcome of the SSP approach is extremely sensitive to the selection of filter bank parameters and can be effective only if the optimum parameters are employed. To obtain them, a brute force search algorithm was utilized to identify the appropriate values by measuring the SNR of the signal’s response while preserving all the axisymmetric features and minimizing the background signals.

To assess the proposed method, two experiments were performed in the lab on eight-inch pipes with the same size and geometry. Each pipe was partially coated with the Denso Tape and had a different size of saw cut defect. The Teletest technology was employed for excitation and receiving the signal. The excitation signal was the Torsional wave mode, T(0,1), a 10-cycle modulated Hann-windowed signal with a couple of different center frequencies around 30 to 35 kHz. These frequencies were selected due to their superior performance before applying the post-processing technique.

A brute force search algorithm was employed to identify the appropriate values for SSP. The results show that the proposed approach with the optimum parameters was successful in enhancing the signal quality. The SSP result was compared experimentally with the traditional approach currently utilized in the Teletest device for UGW inspection and it was established that the proposed SSP technique increased the SNR by approximately 40 dB without distorting the signal. Guided wave technology is still marked as a new technology and any enhancement in terms of signal quality, extending the inspection range, and increasing the sensitivity to small defects will pave the way for the further validation of this technology in the oil and gas industry.

Moreover, it was demonstrated that the proposed method was successful in identifying an 8% CSA saw cut defect that was hidden below the noise level on Pipe A and identifying a 5% CSA saw cut defect on Pipe B that was partially wrapped with the Denso Tape. Moreover, it was successful in enhancing the signal quality and increasing the SNR of both pipes, which can lead to detecting smaller defects and can increase the inspection range. Furthermore, it was particularly successful in removing the background noise (coherent noise) throughout the trace lengths for both pipes, whereas the conventional technique was ineffective. 

## 7. Conclusions

In this work, a novel solution based on split-spectrum processing (SSP), which is an advanced signal processing approach, was proposed to address the problem of coherent noise due to the existence of undesired wave modes that are primarily dispersive in guided wave testing, in particular for coated pipes. The attenuation rates in UGW testing for pipes that are buried or coated are extremely high, which causes a huge reduction in the inspection capability of UGW testing. This is a major limitation of using this technology for the oil and gas industry.

To address this issue, the SSP technique was utilized as a post-processing approach on coated pipes to reduce the attenuation effects. The SSP method, with optimum parameters, utilized data gathered in the laboratory where the pipes were partially coated with Denso Tape for the restoration of signals suffering from attenuation and background noise in order to enhance the signal quality in terms of spatial resolution and SNR. SNR enhancement in UGW testing can improve the ability of the test detect small defects and increase the inspection range. The result illustrated that the SSP algorithm has great potential to decrease the background noise entirely by minimizing the effect of undesired wave modes (background noise) throughout the signal’s trace, whereas the traditional method was not able to do this. 

The conclusions reached in this report will contribute to the development of guided wave testing through more reliable defect detection and signal interoperation for coated and buried pipelines. However, this approach is only validated in limited trials and to make it automated, further field data analysis is required. Hence, further work on this topic will focus on testing more field data with different center frequencies and different types of defects on coated pipes where the attenuation is high and investigating the optimum parameters for these scenarios.
